# Acute Coronary Syndrome after 17 Years of Bare Metal Stent Implantation: “Very” Very Late Stent Thrombosis

**DOI:** 10.1155/2020/9628719

**Published:** 2020-02-06

**Authors:** Raghavendra Rao K, S. Reddy, J. R. Kashyap, K. Vikas, Hithesh Reddy, Vadivelu Ramalingam

**Affiliations:** ^1^Department of Cardiology, Government Medical College and Hospital, Sector 32, Chandigarh 160030, India; ^2^Department of Cardiology, Velammal Medical College Hospital and Research Institute, Madurai 625009, India

## Abstract

Very late stent thrombosis (VLST) is a catastrophic and life-threatening complication after percutaneous coronary intervention which presents as an acute coronary syndrome with significantly high mortality and morbidity. VLST is a rare entity with drug-eluting stents and even rarer with bare metal stents. The exact pathophysiologic mechanism of VLST after BMS implantation is not known although various mechanisms have been proposed. Recently, in-stent neoatherosclerosis with intimal plaque rupture has been proposed as a potential mechanism of VLST after BMS. We report a rare case of VLST occurring 17 years after BMS implantation with angiographic and intravascular imaging evidence which provides insight into the mechanisms of VLST.

## 1. Introduction

Coronary stents are the mainstay of percutaneous coronary interventions and have significantly reduced the rates of restenosis and acute vessel closure [1]. Drug-eluting stents (DES) are preferred over bare metal stents (BMS) since DES significantly reduces in-stent restenosis (ISR) by inhibiting neointimal proliferation. However, stent thrombosis (ST) remains an uncommon but catastrophic complication which usually presents as acute coronary syndrome (ACS) with STEMI (ST elevation myocardial infarction) though it can also present as sudden death, arrhythmias, or acute heart failure [2]. Incidence of stent thrombosis has markedly reduced with the use of dual antiplatelet therapy (DAPT) and adequate optimization of stent implantation [1]. According to the Academic Research Consortium criteria and classification, ST is classified according to the time since stent implantation. Acute ST occurs during the stenting procedure or within the subsequent 24 hours, subacute ST occurs between 1 and 30 days after implantation, late ST occurs between 1 month and 1 year, and very late ST occurs more than 1 year after the procedure [3]. A new term “very (or extreme) very late stent thrombosis (VVLST)” was suggested when ST occurred after five years of stent implantation [2, 4]. Very late stent thrombosis (VLST) occurs more frequently with DES than with BMS, and majority of VLST occurs within 1–4 years of stent implantation. VLST occurring after five years of stent implantation is an exceedingly rare phenomenon, and it is still a rarer entity with BMS [2, 5]. We report a case of “very” very late stent thrombosis occurring 17 years after BMS implantation which presented as acute ST segment elevation myocardial infarction.

## 2. Case Report

A 76-year-old man first reported in the year 2000 with acute-onset retrosternal chest pain of 24-hour duration. Electrocardiogram showed ST segment elevation in the inferior leads with normal sinus rhythm. Apart from diabetes mellitus, other conventional risk factors like obesity, hypertension, smoking, and family history of ischemic heart disease were absent. Routine investigations were within normal limits. Echocardiogram revealed inferior wall hypokinesia with an ejection fraction of 40% with no mitral regurgitation. After receiving a loading dose of aspirin (325 mg), clopidogrel (600 mg), and atorvastatin (80 mg), the patient was taken up for coronary angiography. Coronary angiography revealed a normal left main artery (LM), left circumflex artery (LCX), and left anterior descending artery (LAD). The right coronary artery (RCA) had a significant stenosis in the midsegment, and the patient underwent PCI to RCA with implantation of a bare metal stent (BMS) in the mid-RCA. Drug-eluting stents (DES) were not available at that point of time anywhere in the country. His recovery was uneventful and was discharged on the 4th day on daily aspirin (150 mg), clopidogrel (75 mg), metoprolol (25 mg), atorvastatin (80 mg), and oral hypoglycemic agents. He was on a regular follow-up every 3–6 months since the time of his first coronary intervention. Clopidogrel was stopped after 12 months, and he was advised to continue other medications. The patient remained asymptomatic and was on a regular medical follow-up with no discontinuation of medical therapy at any point of time.

**Figure 1:**
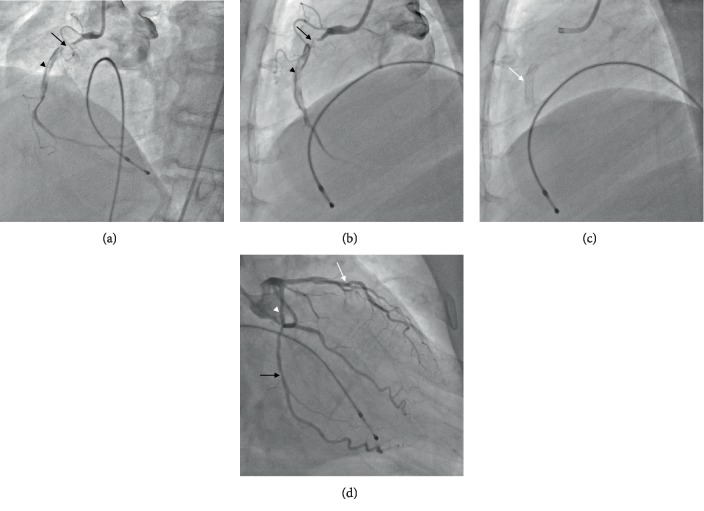
(a) Coronary angiography showing RCA in LAO cranial view with maximum stenosis just proximal to the native bare metal stent (black arrow) and thrombosis within the stent (black arrowhead). (b) RCA in lateral view with 95% stenosis (black arrow) and thrombus inside the stent (black arrowhead). (c) Native bare metal stent (white arrow). (d) RAO caudal view shows plaque in LAD (white arrow) with 30% stenosis in the proximal LCX (white arrowhead) and 50% stenosis in the obtuse marginal artery (black arrow).

**Figure 2:**
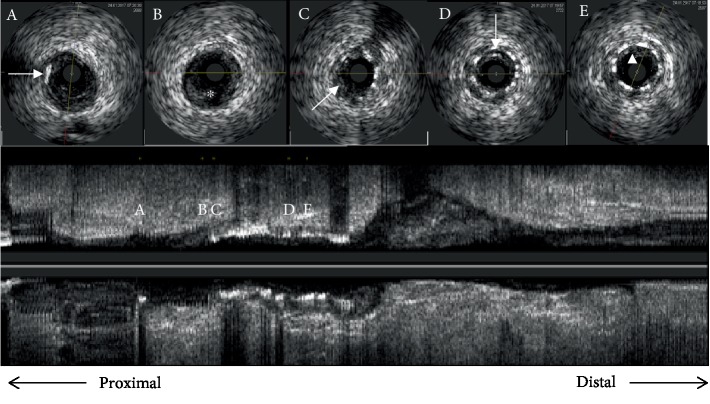
Cross-sectional and longitudinal views: baseline IVUS pullback images from distal to proximal RCA demonstrating (a) an arc of calcium in the proximal RCA (white arrow), (b) a site of plaque rupture at the proximal stent edge (white asterisk), (c) thrombus inside the stent (white arrow), (d) spotty calcification within the stent (white arrow), stent struts (black arrow), and (e) a site of plaque rupture within the stent (white arrowhead).

**Figure 3:**
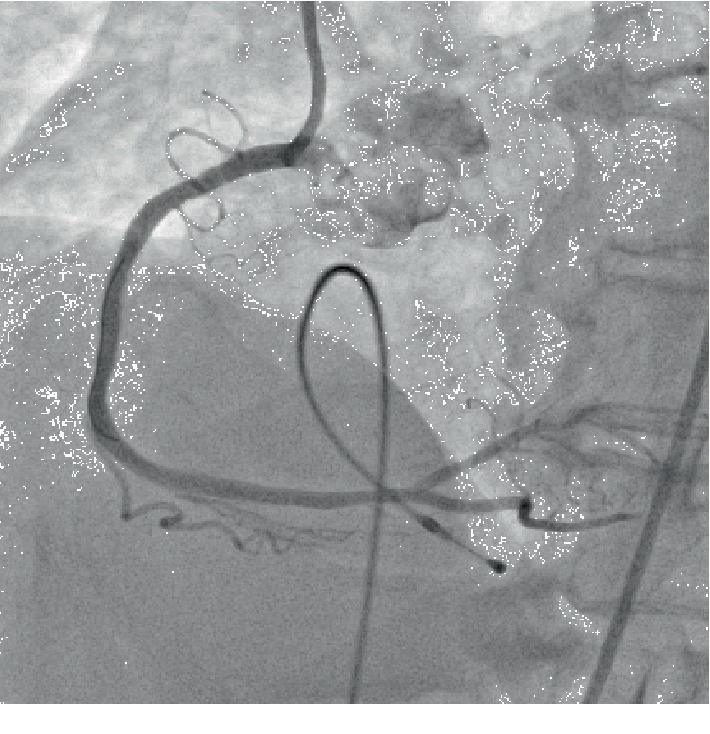
Final angiographic view post-PCI showing adequate stent expansion with good distal opacification.

**Figure 4:**
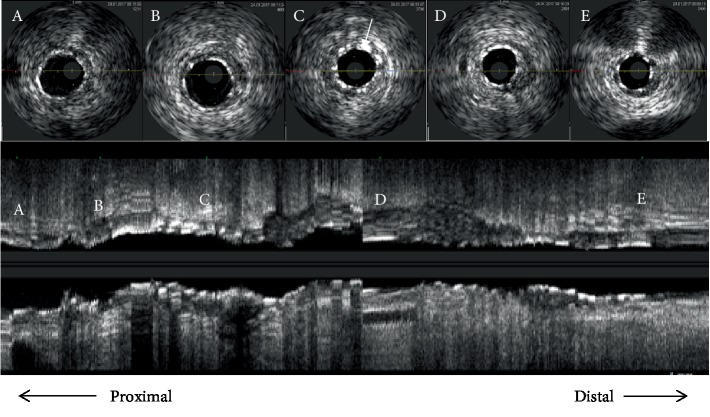
Poststenting IVUS pullback showing adequate stent expansion with an MLD/CSA (minimum lumen diameter/cross-sectional area) of (a) 3.13 mm/8.43 mm^2^ at the proximal RCA, (b) 3.23 mm/8.92 mm^2^ at the previous stent proximal edge, (c) 2.85 mm/6.46 mm^2^ at the stent overlapping region (white arrow), (d) 2.54 mm/5.49 mm^2^ at the distal RCA, and (e) 2.39 mm/4.78 mm^2^ at the very distal RCA.

In January 2017, the patient presented to us with sudden-onset chest pain radiating to the left shoulder of one-hour duration and an episode of syncope. His pulse rate was 40/min regular, and his blood pressure is 90/60 mmHg. Electrocardiogram showed sinus bradycardia with ST elevations in leads II, III, and aVF. The cardiac enzyme troponin T was positive, and echocardiography showed hypokinesia of the inferior wall with no mitral regurgitation and a leftventricular ejection fraction of 45%. Blood sugars were well controlled with normal renal function tests and a hemogram. The patient underwent temporary pacemaker insertion in view of the syncopal episode and bradycardia. Coronary angiography revealed proximal LAD plaque, proximal LCX 30% stenosis, and obtuse marginal 50% stenosis. In proximal RCA 95% stenosis, the mid-RCA stent was thrombus laden extending to the distal RCA. A posterior descending artery (PDA) and posterior left ventricle (PLV) branches were normal (Figures 1(a)–1(d)). He was given loading doses of aspirin (325 mg), clopidogrel (600 mg), and atorvastatin (80 mg) and was taken up for primary PCI to RCA via a right femoral approach. The right coronary artery was engaged with a Judkins right guiding catheter (6 French, 3.5), and the lesion was crossed using a 0.014^″^ BMW guidewire (Balance Middleweight Universal wire, Abbott Vascular, CA, USA). An IVUS (Eagle Eye® Platinum digital IVUS catheter, Volcano Corporation, CA, USA) pullback was taken from distal RCA which showed thrombus extending from proximal to distal RCA. There was plaque rupture at the proximal stent edge and thrombus. Within the stent, there was neoatherosclerosis in the midregion with intimal plaque rupture, spotty calcification, and thrombosis (Figures 2(a)–2(e)). Significant plaque burden and thrombus were also noted in the distal RCA. Eptifibatide (GP IIbIIIa inhibitor) infusion was started, and manual thrombus aspiration was done using a 6 French Thrombuster II® (Kaneka Corporation, Osaka, Japan). The lesion was predilated with a SPRINTER® semicompliant 2.5 × 15 mm balloon (Medtronic, Minneapolis, USA) from distal to proximal RCA at 10-12 atm. The stent Xience Prime (2.75 × 38 mm) (2^nd^-generation everolimus-eluting stent, Abbott Vascular, CA, USA) was deployed in mid-RCA to distal RCA at 10 atm, the second stent Xience Prime (3 × 38 mm) was deployed in proximal RCA to mid-RCA at 12 atm overlapping with the previous stent, and the third stent Xience Prime (3.5 × 28 mm) was deployed from ostial to proximal RCA at 12 atm overlapping with the previous stent. Postdilatation of the distal to ostial RCA stents was done using SPRINTER® noncompliant balloons (Medtronic, Minneapolis, USA) (2.75 × 9 mm, 3 × 12 mm, and 3.5 × 12 mm) successively at 12-18 atm. Postprocedure angiography showed TIMI III flow (Figure 3), and an IVUS pullback was taken which showed good stent strut apposition (Figures 4(a)–4(e)). The patient was discharged in a stable condition on dual antiplatelets and statins.

## 3. Discussion

VLST is an exceedingly rare complication of PCI with recent studies showing an incidence of approximately 0.5% per year with BMS that reaches up to 2% per year with DES. Most cases present as an ST segment elevation myocardial infarction carrying high morbidity and mortality with an annual mortality rates of 10% to 20% [6, 7]. We present a case of “very” very late stent thrombosis occurring 17 years after implantation of a bare metal stent. Our case highlights the importance that the underlying pathophysiologic mechanisms are multifactorial. To the best of our knowledge, this is the first case to report with the longest duration after bare metal stent implantation to thrombosis with an intravascular imaging guidance. Earlier, Acibuca and colleagues reported a case of BMS thrombosis occurring after two decades; however, in their report, intravascular ultrasound (IVUS) was not done and the exact duration was not clear [8]. Our case is unique as IVUS was performed and we demonstrated that multiple factors are responsible for plaque rupture leading to VLST such as (a) persistent peristent strut chronic inflammation leading to plaque rupture at the proximal stent edge, (b) neoatherosclerosis inside the stent which led to plaque rupture, and (c) the presence of calcium suggesting longer duration of the atherosclerotic plaque and subsequent plaque rupture due to a calcified nodule.

Earlier, Sarkees et al. had reported VLST occurring 13 years after BMS implantation which occurred upon termination of antiplatelet therapy [9]. History of discontinuation of antiplatelet therapy was not there in our case. Randomized trials have shown that the incidence of stent thrombosis within the first year of implantation is identical in patients with DES and those with BMS. However, after 1 year, a modest increase is observed in VLST after DES implantation compared to that after BMS implantation [10]. The exact pathophysiologic mechanism for VLST after BMS implantation is not known, but various mechanisms have been proposed. BMS have a rapid endothelialization after implantation which is usually completed by 3 to 6 months unlike DES which takes a longer period; hence, the inflammatory response does not seem to be the triggering cause of VLST with BMS [5, 11, 12].

Of the various hypotheses proposed for VLST after BMS implantation, the most important and plausible mechanism being in-stent neoatherosclerosis and subsequent plaque rupture which presents as an ACS and the histological features are indistinguishable between patients with VLST and patients with ACS unrelated to stent thrombosis [6]. Yumoto et al. showed that VLST after BMS might be caused by a thrombus formation subsequent to a calcified atherosclerotic plaque rupture [11]. Stent strut malapposition and positive arterial remodeling and late acquired stent malapposition have also been suggested as probable etiologies for VLST after BMS [13]. Late stent malapposition occurs with both DES and BMS though it is more common after DES implantation and increases the risk of VLST [12]. In the present case, there was no clear IVUS evidence of stent malapposition. Metallic stent struts being a foreign body can induce persistent peristrut chronic inflammation which can accelerate atherosclerotic changes and subsequent plaque rupture [14]. Long-term follow-up studies using intravascular imaging guidance after BMS implantation have shown that the neointima transforms into lipid-laden tissue with narrowing of the lumen, expansion of the neovascularization into the intima, calcification, and advanced atherosclerotic changes like intimal disruption with thrombus formation leading to ACS [15]. Our case showed the presence of calcium in both the native vessel and the stented segment along with evidence of neointimal rupture, plaque rupture at the proximal stent edge possibly due to peristent strut inflammation, and superimposed thrombus formation.

## 4. Conclusion

Based on our case study and IVUS analysis, we conclude that multiple mechanisms contribute to VLST after BMS implantation predominantly due to neoatherosclerotic plaque rupture, peristent strut chronic inflammation leading to plaque rupture, and calcific atherosclerotic plaque rupture. All the above mechanisms either alone or in combination may lead to plaque rupture eventually causing acute coronary syndrome.
